# Zinc oxide nanoparticles from *Cassia auriculata* flowers showed the potent antimicrobial and *in vitro* anticancer activity against the osteosarcoma MG-63 cells

**DOI:** 10.1016/j.sjbs.2021.04.001

**Published:** 2021-04-12

**Authors:** Vidya Devanathadesikan Seshadri

**Affiliations:** Department of Pharmacology & Toxicology, College of Pharmacy, Prince Sattam Bin Abdul Aziz University, Al–Kharj, Saudi Arabia

**Keywords:** Zinc nanoparticles, Osteosarcoma, *Cassia auriculata*, Apoptosis, MG-63 cells

## Abstract

Osteosarcoma (OS) is a foremost mesenchymal bone neoplasm and it can occur at any age with survival rate is nearly 2–8 times lesser in elders than in teenagers. The clinical therapies for cancer treatment have gradually becoming outdated because of the developments of nano-medicine and multi-targeted drug-delivery. In this work, we green synthesized the zinc oxide nanoparticles from the *Cassia auriculata* flower (AS-ZnONPs) extract and evaluated its antimicrobial and *in vitro* anticancer potential against the OS MG-63 cells. The synthesized AS-ZnONPs were confirmed and characterized by using UV–vis spectroscopy, XRD, FE-SEM, and photoluminescence techniques. The antimicrobial activity of AS-ZnONPs was studied by disc diffusion technique. The viability of AS-ZnONPs treated MG-63 cells were examined by MTT assay. The apoptotic cells in the AS-ZnONPs treated MG-63 cells were assayed by dual staining. The MMP status of AS-ZnONPs treated cells were tested by Rh-123 staining. The cell adhesion assay was performed to detect the anticancer effects of AS-ZnONPs against MG-63 cells. The results of UV–vis spectroscopy, XRD, FE-SEM, and photoluminescence techniques proved the formation of AS-ZnONPs and it has the hexagonal wurtzite structures. AS-ZnONPs displayed the potent antimicrobial activity against the tested microbial strains. The AS-ZnONPs were appreciably inhibited the cell viability of MG-63 cells. The outcomes of fluorescence staining proved that AS-ZnONPs reduced the MMP and prompted the apoptosis in MG-63 cells. In conclusion, our discoveries demonstrated that the formulated AS-ZnONPs has the potent antimicrobial and *in vitro* anticancer activity against the MG-63 cells. The AS-ZnONPs could be potent chemotherapeutic agent in the future to treat the OS.

## 1 Introduction

Osteosarcoma (OS) is a foremost mesenchymal bone neoplasm distinctive for a pediatric population and it can occur at any age group. Regrettably, the survival rate of aged victims are nearly 2–8 times lower that the teenagers. OS can quickly contribute distant metastasis and in most cases the disease are in advanced stage at diagnosis. Lungs are the utmost site of OS metastasis (Luetke et al., 2014, Corre et al., 2020). Nearly 40–50% of OS tumors are chemoresistant. Numerous processes are reported recognized in the multidrug resistance of cancer cells e.g. efflux pumps, elevated detoxification, reduced drug utilization and augmentation of DNA repair mechanisms. Hence, the result of the treatments are often deprived with a 5-year survival are 55% (Muley et al., 2020, Susa et al., 2009). Hence, the exploration for the novel approaches to treat the OS is essential nowadays.

The microbial diseases are the critical health problem, which considered as the imperative cause of mortality worldwide. The elevated infections and occurrences of pathogens, emergence of antibiotic resistance, novel bacterial mutations, less specified antibiotics in underdeveloped countries, and hospital borne infections are the global health problem to humans, especially children (da Silva et al., 2019). The rise of antibiotic resistant pathogens is the biggest cause of numerous infections that was regarded as severe public health hazards, which makes the elevated mortality worldwide. The augmented antibiotic resistance of microbes is the imperative health threat to the peoples across the world; hence there is a huge need to explore the novel approaches to overcome this issue. The nanoparticles (NPs) signify the promising solution to overcome this issue of microbial resistance (Geffers and Gastmeier, 2011).

The nanotechnology combined with biology plays a huge role in the creation of more efficient anticancer and antimicrobial agents. Metal oxide NPs offer talented and wide standpoints for biomedical area via the generation of nanomaterials, anticancer and antimicrobial agents, cell imaging, biosensing, and gene delivery (Mishra et al., 2017). The NPs exhibit the exclusive nature of having a huge surface to volume ration, which builds them as more appropriate agent in the application oriented performances (Smith et al., 2013).

The zinc oxide nanoparticles (ZnONPs) are easier to generate, non-toxic, eco-friendly, bio-compatible, and bio-safe that makes theme as potential biological application (Rao et al., 2020). NPs as a drug delivery agents have a numerous like augmented efficiency, decreased toxicity, fewer cost, and efficacy against the multidrug resistance cancer (Steckiewicz and Inkielewicz-Stepniak, 2020). Currently, the NPs are widely formulated through chemical and physical techniques. Furthermore, the NPs attained with these techniques unstable and toxic, which makes them less efficient (Magudieshwaran et al., 2019). Consequently, green synthesis approach, which consider as a less toxic and safe technique was widely explored in recent times. This greener approach utilizes numerous biological resources due to the existence of great secondary metabolites, which could be a perfect reduction and stabilization agents of NPs (Dutta et al., 2016). The ZnONPs appeared to be more potent against numerous diseases and the anticancer activities of ZnONPs was previously reported (Bisht and Rayamajhi, 2016).

*Cassia auriculata*, a shrub belongs to the Caesalpiniaceae family, which is extensively utilized for the treatment of diabetes, conjunctivitis, rheumatism, and as tonic (Nille and Ramachandra Reddy, 2016). Moreover, the biological effects of *C. auriculata* like urinary disorders, skin diseases, ulcers, and fever was also reported previously (Kainsa et al., 2012). It was already reported that the plant *C. auriculata* is an excellent source of metallic nanoparticles (Prasad et al., 2020, Kumar et al., 2011). But the formulation of ZnONPs from the *C. auriculata* flowers and its antimicrobial and anticancer potential was not addressed yet. Hence, in this current investigation we formulated the ZnONPs from the *C. auriculata* flowers (CA-ZnONPs) and examined its antimicrobial against pathogens and *in vitro* anticancer potential against the osteosarcoma MG-63 cells.

## 2 Materials and methods

### 2.1 Chemicals

Zinc nitrate (Zn (NO_3_)_2_·6H_2_O), 3-(4,5-dimethylthiazol-2-yl)-2,5-diphenyl tetrazolium bromide (MTT), phosphate buffered saline (PBS), and Acridine orange/Ethidium bromide (AO/EB), and other chemicals were attained from Sigma Aldrich, USA.

### 2.2 Collection of plant material and synthesis of ZnONPs

Initially, the *C. auriculata* flower was collected from the Bharathidasan University, Trichirappalli, and then flower was washed three times with distilled water and let it dried under shade condition. Then, 10 g of fresh *C. auriculata* flower was mixed with 100 mL of distilled water and heat-macerated at 80 °C for 20 min. The resultant suspension was filtered using Whatman No.1 filter paper. 0.1 M of (Zn (NO_3_)_2_·6H_2_O) solute was mixed with 100 mL of *C. auriculata* flower extract and stirred continually at a temperature of 80 °C for 4–6 h. After that solution will become completely dried and the dried CA-ZnONPs were collected and the resultant NPs were calcined at 700 °C for 5 h.

### 2.3 Characterization of synthesized CA-ZnONPs

The CA-ZnONPs was characterized by an X-ray diffractometer (model: X’PERT PRO PANalytical). The lattice constants ‘a’ and ‘c’ of wurtzite structure can be calculated by using the relation (Suryanarayana and Norton, 1998).

$$\frac{1}{d^{2}}=\frac{4}{3}\left(\frac{h^{2}+hk+k^{2}}{a^{2}}\right)+\frac{l^{2}}{c^{2}}$$

With the first order approximation (n = 1) for the (1 0 0) plane, the lattice constant ‘a’ is obtained through the relation a = $\frac{\lambda }{\sqrt{3}sin\theta }$and lattice constant ‘c’ is derived for the plane (0 0 2) by the relation $c=\frac{\lambda }{sin\theta }$. For CA-ZnONPs, values of the lattice parameters ‘a’ and ‘c’ are estimated 3.256 Å and 5.215 Å respectively. The size of the NPs was determined by the Scherrer formula (Muhammad et al., 2019),

$$Average\;crystal\;size\;D=\frac{k\lambda }{\beta_{D\;cos\theta }}$$

where D-is the size (nm), λ is the radiation wavelength (1.5406 Å for CuKα), k is a constant (0.94), β_D_-is the peak width at half-maximum in radian along (1 0 1) plane and is Bragg’s diffraction angle.

The diffraction arrays were noted in the range of 20–80° for the ZnO, where the monochromatic wavelength of 1.54 Å was utilized. Field Emission Scanning Electron Microscopy (FE-SEM) analysis is one of the promising techniques for the topography study of the sample. The CA-ZnONPs was analyzed by FE-SEM (Carl Zeiss Ultra 55 FESEM) with EDAX (model: Inca). UV–Visible spectroscopy was performed on a spectrophotometer (Perkin Elmer Instrument, USA). Photoluminescence spectra was determined using the Cary Eclipse spectrometer (Xiong et al., 2007).

### 2.4 Antimicrobial assay

The CA-ZnONPs was tested towards Gram-positive (*Staphylococcus aureus* and *Streptococcus pneumoniae*) and Gram negative (*Escherichia coli* and *Klebsiella pneumonia*) strains with the amoxicillin as standard. The 40 mg of green synthesized CA-ZnONPs were dispersed into 1 mL of sterile 5% Dimethyl sulfoxide (DMSO) using for stock solution. The antimicrobial activity was carried out by the well diffusion method. The CA-ZnONPs was tested against gram-positive (*S. aureus* and *S. pneumonia*), gram-negative (*K. pneumonia* and *E. coli*) strains on Mueller Hinton Agar (MHA) as reported by the Clinical and Laboratory Standards Institute (CLSI) (Wright, 2000). The antimicrobial study was conducted at a doses of 1, 1.5, and 2 mg/mL of the CA-ZnONPs was loaded on the strains-seeded plates. Then plates were maintained at 37˚C for 24 h. The zone inhibition near the wells were monitored and noted. For positive control, standard antibiotic Amoxicillin (30 µg disc) was used.

### 2.5 Collection and maintenance of cell culture

OS MG-63 cells were acquired from the American Type Culture Collection (ATCC), USA and sustained in DMEM medium along with FBS (10%) and Penicillin/Streptomycin (1%) at 37 °C in a dampened chamber with CO_2_ (5%) and air incubation (95%).

### 2.6 MTT cytotoxicity assay

The cytotoxic effects of fabricated CA-ZnONPs was examined by MTT assay. For this, MG-63 cells was loaded onto the 96-well plate at 6 × 10^3^ cells/well density and incubated for 24 h at 37 °C. Then the plate was loaded with the diverse doses (5–35 µg) of fabricated CA-ZnONPs, and again incubated for 24 h at 37 °C. After the 24 h incubation, 100 μl of MTT solution was mixed to every well and then plates were again incubated for 4 h. Then medium was eliminated from each well and 100 μl of serum-free medium were refilled and developed formazan crystals were liquefied through administering the DMSO. Lastly, the absorbance was measured by microplate reader at 570 nm. The morphological appearances of CA-ZnONPs (15 and 20 µg) treated MG-63 cells were examined beneath the light microscope to detect the CA-ZnONPs provoked morphological changes in MG-63 cells.

### 2.7 Cell adhesion assay

The MG-63 cells were placed on the 96-well plate and maintained for 24 h at 37 °C. Then cells were treated with the 15 and 20 µg of fabricated CA-ZnONPs and maintained for 24 h in 5% CO_2_ incubator at 37 °C. After that cells were cleansed with saline and stained with tryphan blue to identify the dead and viable cells. The tryphan blue stains the dead cells and the viable cells remain unstained. MG-63 cells were examined beneath the light microscope and the numbers dead and viable cells were detected.

### 2.8 Dual staining assay

The dual staining (AO/EB) assay was implemented to investigate the early and late stage apoptotic cell death in the CA-ZnONPs administered MG-63 cells. Shortly, the fabricated CA-ZnONPs were supplemented (15 and 20 µg) to the MG-63 cells and then were incubated for 24hrs. Later, cells were stained with dual stain AO/EB for 30mins. Lastly, cells were examined beneath the fluorescent microscope to identify apoptotic cell death in the MG-63 cells.

### 2.9 Measurement of mitochondrial membrane potential (MMP)

The level of MMP in the CA-ZnONPs supplemented MG-63 cells were investigated via the Rhodamine-123 (Rh-123) staining. Briefly, the MG-63 cells was grown in 6-wellplate with the varied doses of formulated CA-ZnONPs (15 and 20 µg). The CA-ZnONPs treated MG-63 cells were then stained with the RH-123 fluorescent stain and incubated for 30 min at 37 °C. Lastly the alterations in the MMP was examined under fluorescent microscope.

### 2.10 Statistical analysis

Data were studied by using the SPSS software. Results were depicted as mean ± SD of triplicates. The ANOVA successively DMRT test was implemented to analyze the variations between groups. *P-*value less than 0.05 noticed as significant.

## 3 Results

### 3.1 Characterization of synthesized CA-ZnONPs

XRD patterns of formulated CA-ZnONPs were showed in Fig. 2. The XRD peaks are located at angles (2θ) of 31.72, 34.39, 36.21, 47.47, 56.55, 62.87, 66.40, 67.94, 69.17, 72.55 and 77.02 corresponds to (1 0 0), (0 0 2), (1 0 1), (1 0 2), (1 1 0), (1 0 3), (2 0 0) (1 1 2), (2 0 1), (0 0 4), and (2 0 2) planes of the CA-ZnONPs, respectively. The CA-ZnONPs was confirmed that the hexagonal phase with wurtzite structure and which is confirmed by the JCPDS data 79-2205. The ZnO NPs average particle size is 41.25 nm.

**Fig. 1:**
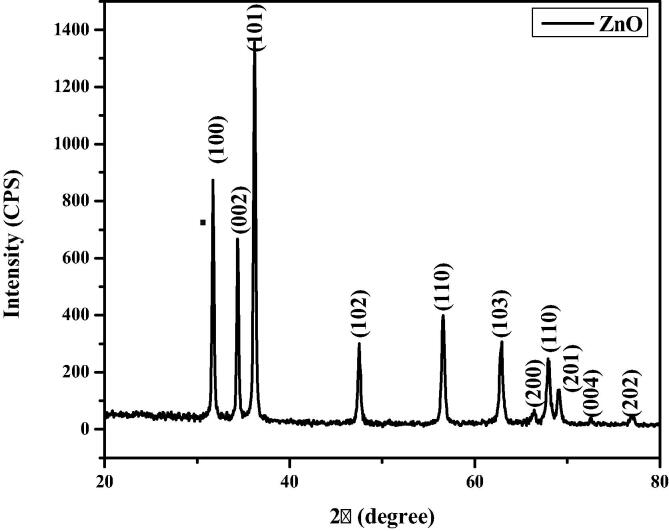
XRD patterns of synthesized CA-ZnONPs.

**Fig. 2:**
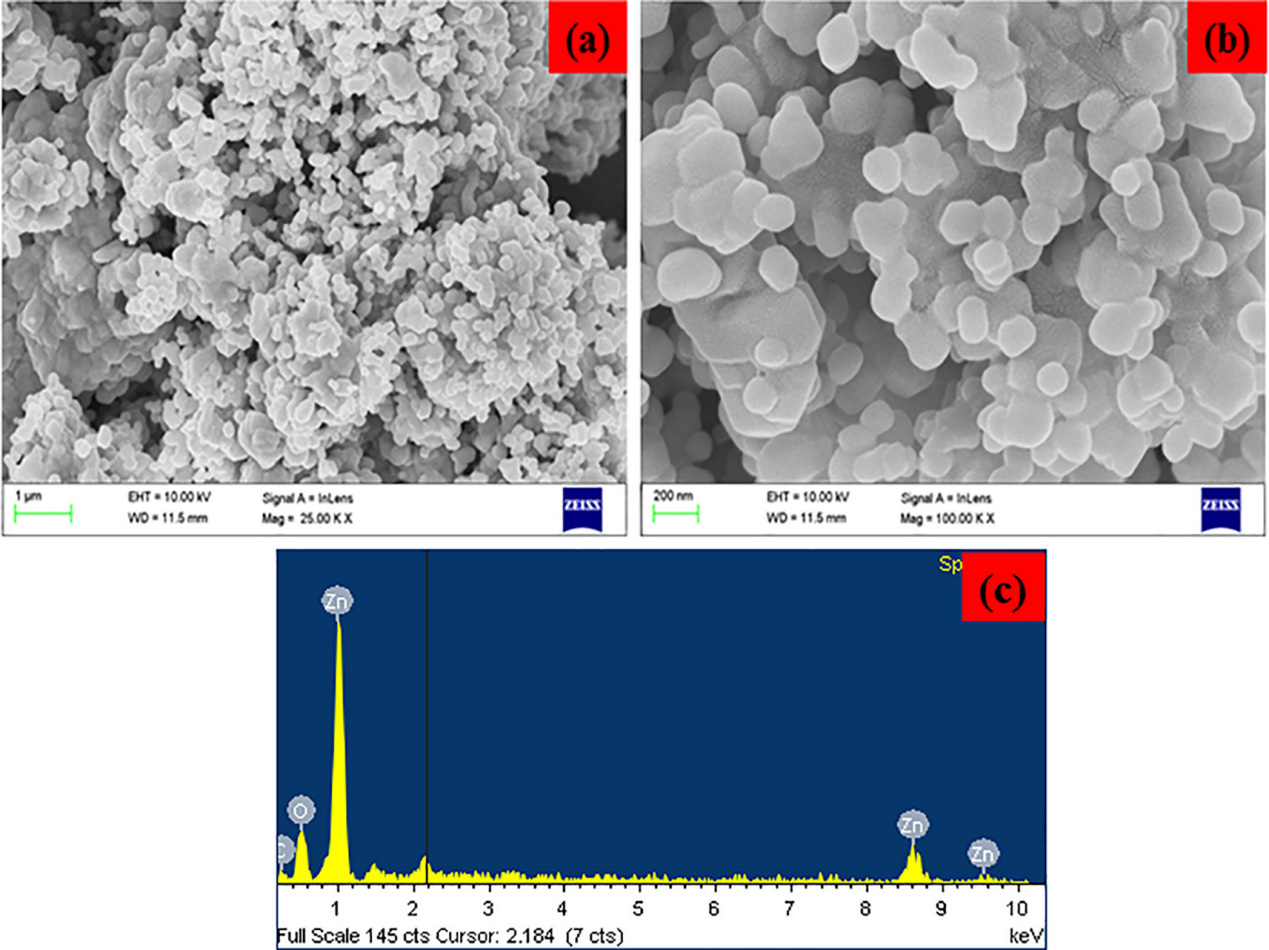
(a-b). FE-SEM images and (c) EDAX spectra of synthesized CA-ZnONPs.

The surface morphology of the formulated CA-ZnONPs using *C. auriculata* flower extract is shown in Fig. 2(a-b). The CA-ZnONPs are exhibit a flake structure and the average size of the CA-ZnONPs is 41 nm. EDAX spectra of the CA-ZnONPs is displayed in Fig. 2c. From the EDAX study of CA-ZnONPs, the chemical constituents like Zn, O, and C are found to be 35.28%, 36.53%, and 28.19% respectively.

**Fig. 3:**
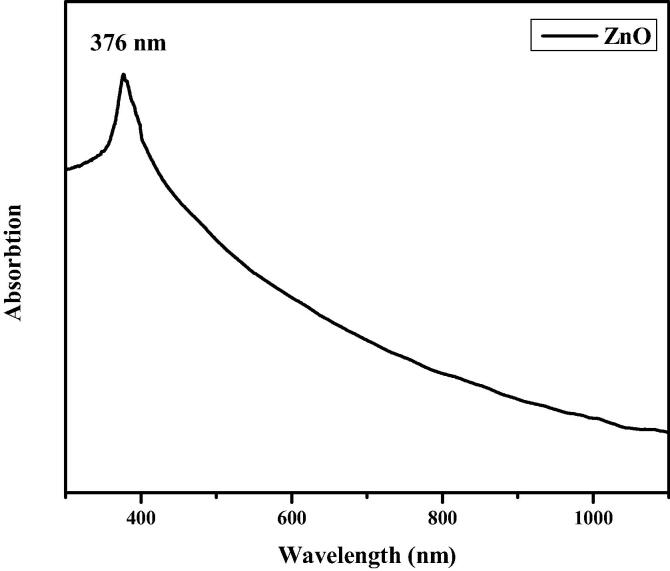
UV–visible spectrum of synthesized CA-ZnONPs.

**Fig. 4:**
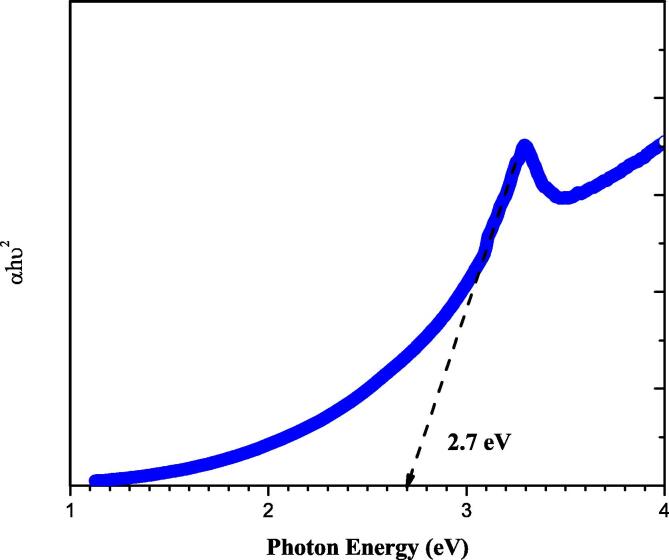
Bandgap values of synthesized CA-ZnONPs.

Fig. 3 demonstrates the UV–visible spectrum of fabricated CA-ZnONPs. The CA-ZnONPs absorption sharp peak is observed at 376 nm, which belief to arise from the near band edge free excitons. Fig. 4 indicated that, the small red-shift (0.67 eV from standard bulk band gap at room temperature) observed for green synthesized CA-ZnONPs (2.7 eV) as compared to bulk ZnO (3.37 eV).

**Fig. 5:**
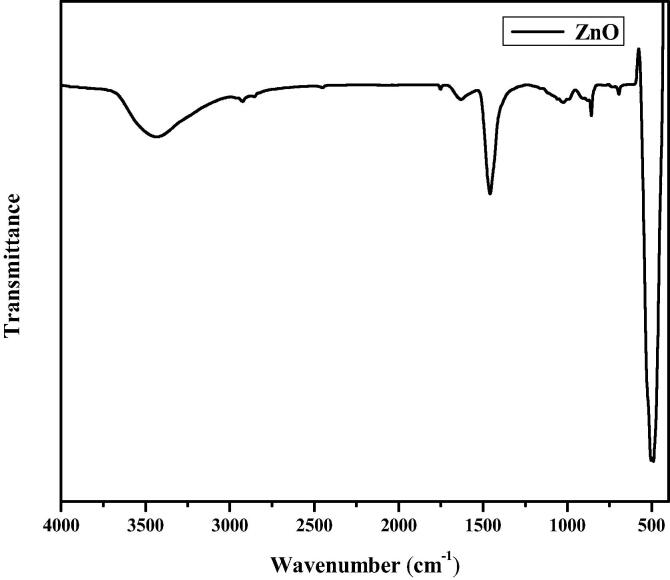
FT-IR analysis of synthesized CA-ZnONPs.

The FT-IR spectrum of CA-ZnONPs is illustrated in Fig. 5. The broad -OH hydrogen bonds were noted at 3441 and 1632 cm^−1^ for CA-ZnONPs. The C–H asymmetric stretching bond is found at 2991 cm^−1^. The stretching frequency of Zn-O is center at 1453 cm^−1^ for the CA-ZnONPs. The Zn-O stretching band is monitored at 472 cm^−1^ for CA-ZnONPs.

**Fig. 6:**
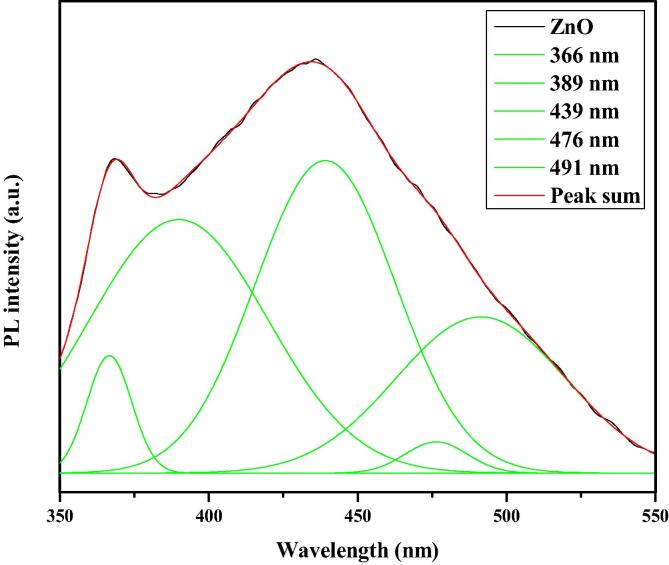
PL spectrum of synthesized CA-ZnONPs.

The PL spectrum of CA-ZnONPs using *Cassia auriculata* flower extract exciting wavelength of 325 nm at 37 °C is illustrated in Fig. 6. The PL spectrum of the ZnO NPs sample values is observed at 363, 393, 407, 429, 466, and 489 nm, as shown in Fig. 6. From the optical properties of ZnO could be established from PL spectrum, the information about the crystal modality, structural defects like oxygen vacancy, Zn interstitials, surface defects, etc. The UV radiations (366 and 389 nm) are usually credited to the near band edge (NBE) emission due to free exciton recombination. The blue and blue-green emission bands (439, 476, and 491 nm) are attributed to singly ionized Zn vacancies and interstitial oxygen vacancies.

### 3.2 Antimicrobial activity of synthesized CA-ZnONPs

**Fig. 7:**
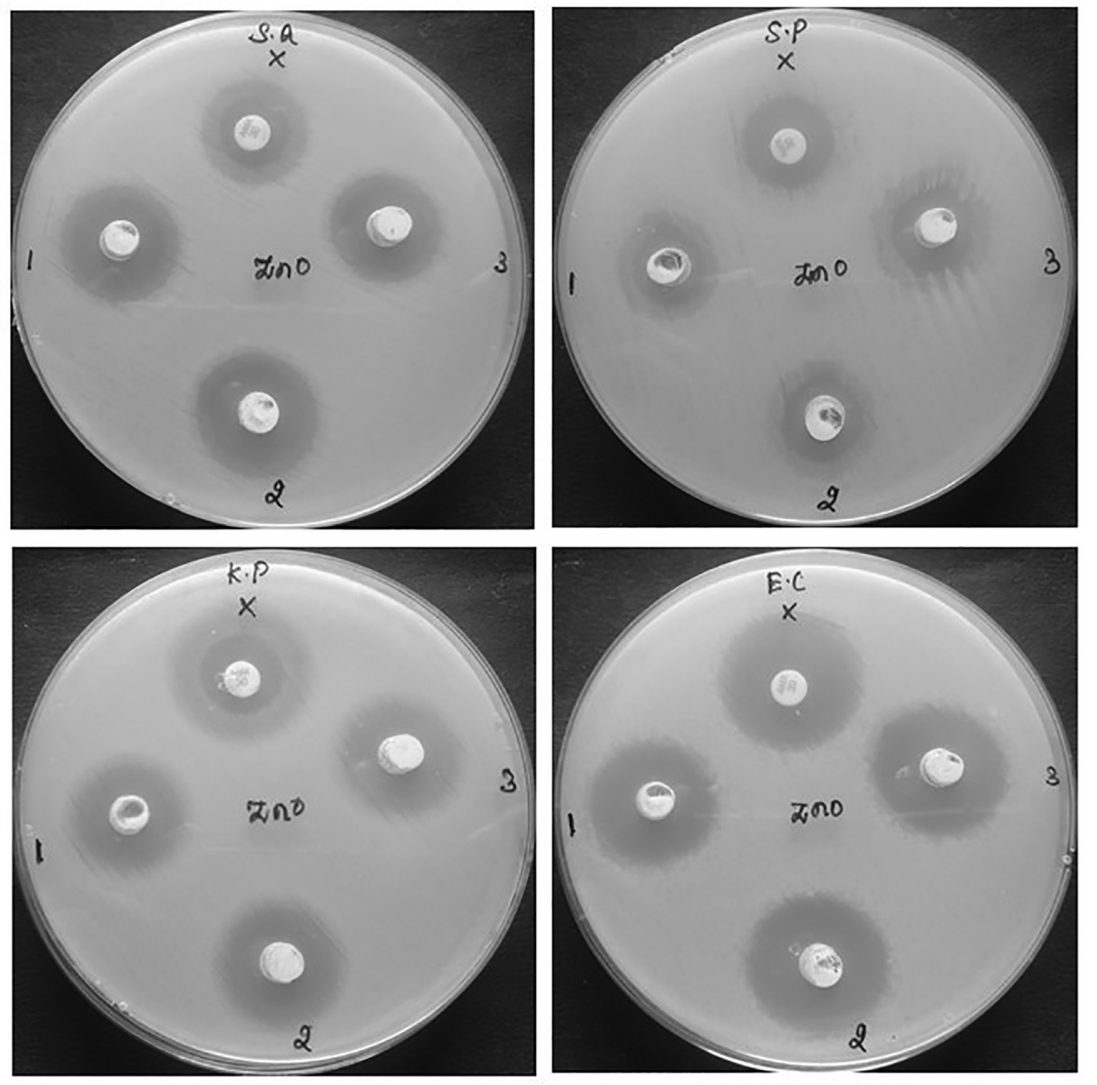
Antimicrobial activity of the synthesized CA-ZnONPs. The CA-ZnONPs treated strains demonstrated the clear inhibition zones around the treatment discs with different concentrations (1000, 1500 and 2000 μg/mL) of formulated CA-ZnONPs. When compared with other strains, *E. coli* exhibited the more sensitivity against the CA-ZnONPs.

The antimicrobial activity of CA-ZnONPs was investigated against the gram-positive (*S. aureus* and *S. pneumonia*) and gram-negative strain (*K. pneumonia* and *E. coli*) strains by the well diffusion method (Fig. 7). The antimicrobial activity of CA-ZnONPs showed zones of inhibition around the well loaded with different concentrations (1000, 1500 and 2000 μg/mL) of formulated CA-ZnONPs. The zone size observed, ranged from 18 mm to 25 mm, based on the concentration of formulated CA-ZnONPs and the nature of the test organisms. The *E. coli* bacteria showed the more sensitivity against the CA-ZnONPs than other strains.

### 3.3 Effect of synthesized CA-ZnONPs on the cell morphology and viability of MG-63 cells

**Fig. 8:**
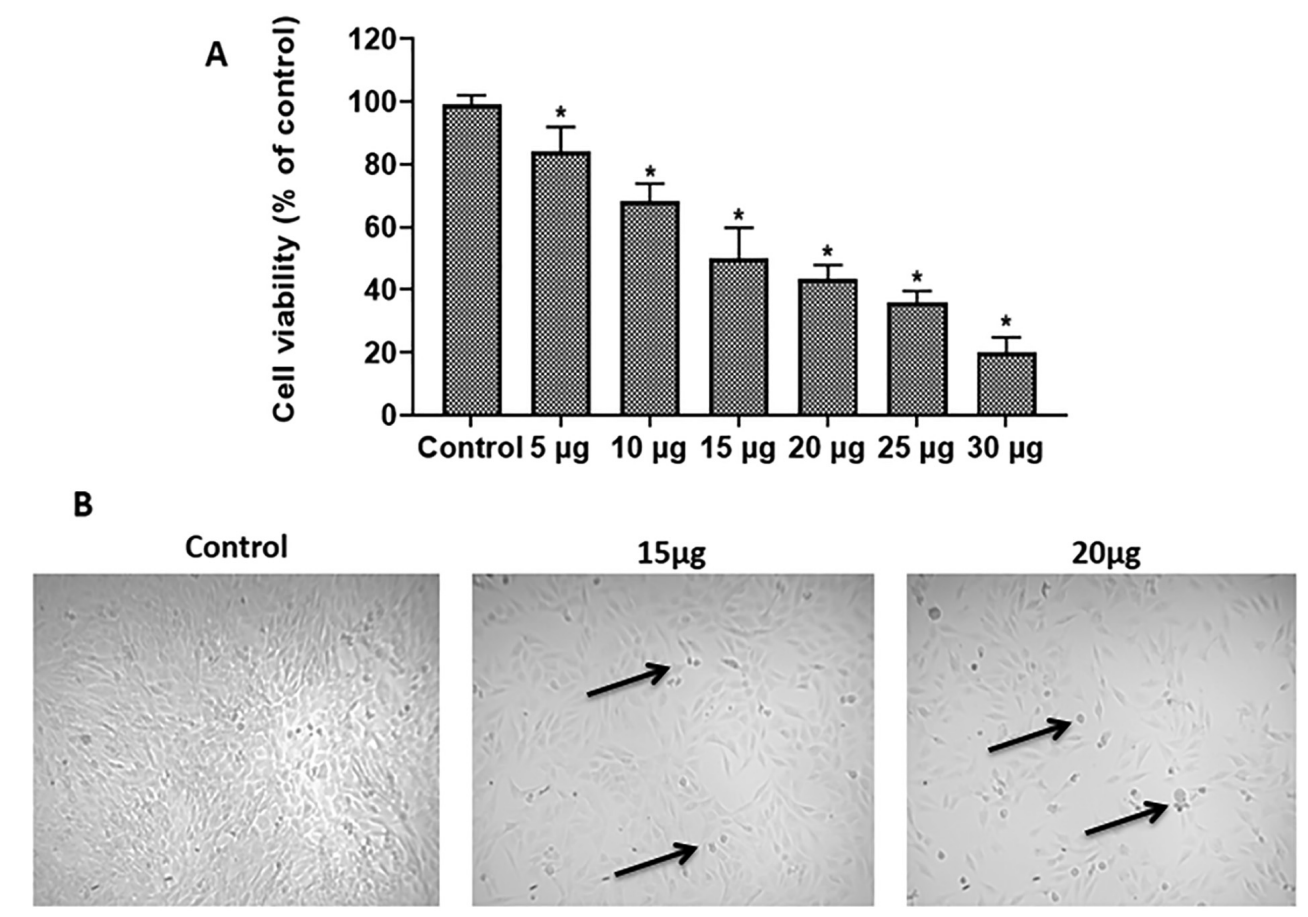
Effect of synthesized CA-ZnONPs on the cell viability and morphological changes of MG-63 cells. Data were illustrated as mean ± SD of triplicate values. Significance was determined via ANOVA successively DMRT study; note: ‘*’ p < 0.05 while comparing with control. Fig. 1B shows the morphological changes of CA-ZnONPs treated MG-63 cells. The morphological alterations like shrinkage and detachments were pointed out by using arrow marks.

The cytotoxic properties of formulated CA-ZnONPs against the MG-63 cells was inspected by MTT assay. As displayed in the Fig. 8A, the CA-ZnONPs was notably repressed the viability of MG-63 cells at dose reliant manner. The increased doses of CA-ZnONPs exhibited the reduced viability of MG-63 cells. The diverse dosages (5–35 µg) of formulated CA-ZnONPs tested against the MG-63 cells and among them 20 µg of 5–35 µg displayed the 50% of cell growth inhibition. Hence, 20 µg of CA-ZnONPs was opted as IC_50_, thereby 20 µg and 15 µg (low dose) of CA-ZnONPs was chosen for the additional studies.

The morphological observations of CA-ZnONPs administered MG-63 cells disclosed the huge alterations of MG-63 cell morphology. A vast alteration was detected on the morphology of CA-ZnONPs treated MG-63 cells than the control. The cell shrinkages, rounding, unequal shape, and cell detachments were noted on the 15 and 20 µg of CA-ZnONPs administered MG-63 cells (Fig. 8B).

### 3.4 Effect of CA-ZnONPs on the MMP level of MG-63 cells

**Fig. 9:**
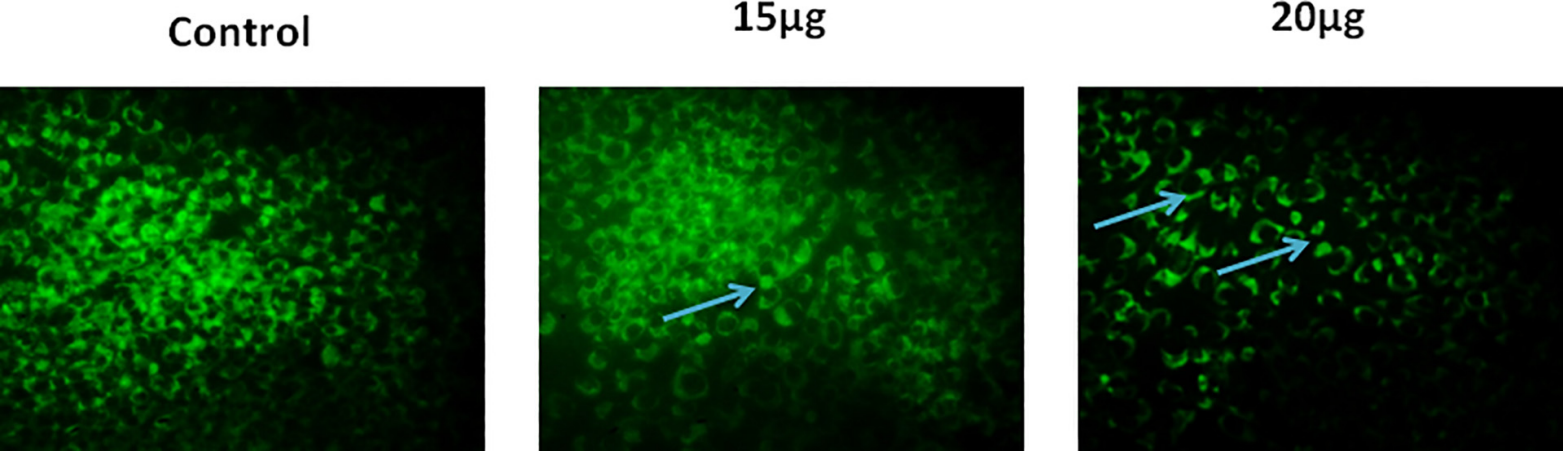
Effect of synthesized CA-ZnONPs on the MMP level of MG-63 cells. The control cells illustrating the strong green fluorescence that indicates the normal MMP, whereas, the 15 and 20μf of formulated CA-ZnONPs supplemented MG-63 cells revealing the reduced green fluorescence that indicating the loss of MMP (blue arrows) in the MG-63 cells.

The CA-ZnONPs treatment to the MG-63 cells displayed the drastic reduction in the MMP status, which is identified by Rh-123 staining. The increased MMP status of cells displays the bright green fluorescence and deteriorated MMP of cells indicates the disaggregated monomer cells, which displays the dull green fluorescence. As illustrated in the Fig. 9, control cells demonstrated the high green fluorescence, contrastingly, CA-ZnONPs administered MG-63 cells exhibited the remarkably dull green fluorescence. This outcome evidenced that the CA-ZnONPs was effectively reduced the MMP of MG-63 cells.

### 3.5 Effect of CA-ZnONPs on the apoptotic cell death of MG-63 cells

**Fig. 10:**
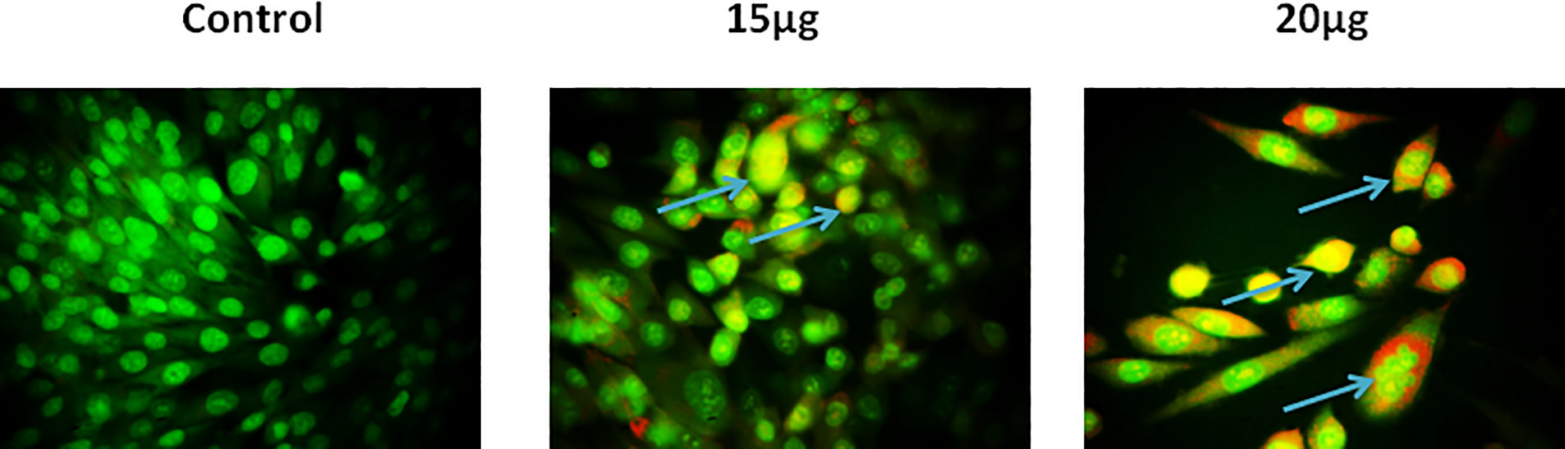
Effect of synthesized CA-ZnONPs on the apoptotic cell death in MG-63 cells. The control cells exhibiting the green fluorescence, contrastingly the formulated CA-ZnONPs (15 and 20 µg) administered MG-63 cells exhibited the strong orange/red fluorescence that proves the apoptotic cell death (blue arrows).

Fig. 10 evidenced that the CA-ZnONPs was effectively stimulated the apoptotic cell death on the MG-63 cells that were proved by dual (AO/EB) staining assay. AO/EB staining technique was executed to investigate the nuclear damages during the apoptotic cell death. The control cells exhibited the green fluorescence, contrastingly the CA-ZnONPs (15 and 20 µg) administered MG-63 cells exhibited the strong orange/red fluorescence, which exhibits the early and late apoptotic cell death. This outcome evidenced the apoptotic inducing potential of formulated CA-ZnONPs on the MG-63 cells. The CA-ZnONPs treatment was stimulated the nuclear damages thereby apoptotic cell death.

### 3.6 Effect of CA-ZnONPs on cell adhesion of MG-63 cells

**Fig. 11:**
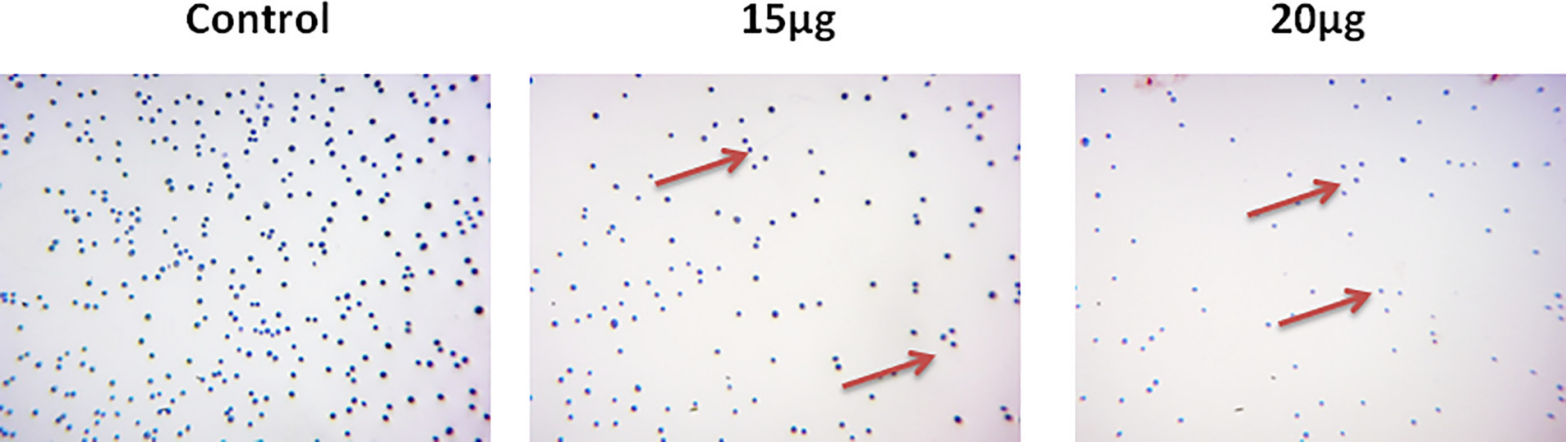
Effect of synthesized CA-ZnONPs on the cell adhesion of MG-63 cells. Shows that the 15 and 20 µg of fabricated CA-ZnONPs treated MG-63 cells demonstrated the reduced cell adhesion (brown arrows).

Fig. 11 displayed that the formulated CA-ZnONPs effectively repressed the cell adhesion of MG-63 cells. The control cells displayed the augmented cell adhesion, contrastingly 15 and 20 µg of fabricated CA-ZnONPs treated MG-63 cells were demonstrated the suppressed cell adhesion. These outcomes evidenced that the formulated CA-ZnONPs could disaggregate the cells through the inhibition of cell adhesion of MG-63 cells (Fig. 11).

## 4 Discussion

OS is an imperative malignant bone tumor in child and juveniles. Moreover, the customary care for OS patients like surgical resection along with the general chemotherapy has the 5 year survival rate of 70% (Chen et al., 2013). The exact mechanisms of OS pathogenesis are not yet fully elucidated yet. The OS treatment often failed because of the emergence of resistance to chemotherapy and increased metastasis (Harrison et al., 2018, Basile et al., 2020). The exclusive features of the NPs like huge surface area, mechanical strength, and stability make the NPs hugely well-matched for numerous pharmacological utilizations like cancer, therapy, antimicrobials, and targeted drug delivery. The remarkable possessions of the NPs are hold both the hydrophobic and hydrophilic drugs that makes NPs more appropriate drug carriers (Thanh and Green, 2010). The types and properties of the metals utilized for the NPs formulation primarily defines the NPs final use. Numerous metals like copper, silver, gold, and many others were extensively utilized for the green formulation of NPs by using plant sources (Mittal et al., 2013). Among them, the ZnO is an inorganic compound that rarely exists in the nature. It exists in the form of crystalline and the manganese impurities in its structure gives slight orange/red color appearance to the naturally occurring ZnO. The ZnO was reported to responsible for the numerous biological activities (Sharma et al., 2020).

The formulation of metal oxide NPs using plant sources holds the recent trend of an eco-friendly approach via minimizing the dangerous toxic chemicals. In this exploration, CA-ZnONPs was fabricated by the green route using *C. auriculata* flower extract. The CA-ZnONPs were characterized by various techniques. The XRD pattern revealed that the CA-ZnONPs exhibits a hexagonal wurtzite structure (Fig. 1). Nanoflake-like morphology was noted in FESEM study (Fig. 2). Fig. 4 demonstrated the small red-shift (0.67 eV from standard bulk band gap at 37 °C) for green synthesized CA-ZnONPs (2.7 eV) as compared to bulk ZnO (3.37 eV). Whereas ZnO NPs results in a decrease to diverge from the Burstein-Moss shift (Lu et al., 2007, Banerjee et al., 2010), the estimated band gap value is close to the bulk ZnO, and this results showed that no indication of quantum size effect (Yogamalar and Bose, 2011).

Apoptosis is a cellular physiological process that is regulated through two imperative cascades i.e. intrinsic (mitochondrial-originated) apoptotic pathway and extrinsic (death receptor-regulated) apoptotic pathway. Mitochondria contributes an essential function in apoptosis through discharging the cytochrome *c* into the cytoplasm that further leads to the stimulation of the caspase-dependant pathways (Antico Arciuch et al., 2012). The flaws in apoptosis contributes an imperative role in the tumor progression. The cytotoxic effects of numerous antineoplastic drugs are commonly convoyed by an augmented apoptosis (Hassan et al., 2014). The metastatic OS cells has numerous overt characteristics, which makes them a less vulnerable to the existing chemotherapeutic drugs like cisplatin, methotrexate, and Adriamycin. Nonetheless these drugs primarily target the non-metastatic OS cells (Luetke et al., 2014). Hence, there is an urgent need for the exploration of novel sources to treat the metastatic and recurrent OS. Consequently, the prompting of apoptosis in cancer cells could enhance the possibilities of successful chemotherapy to counteract the cancer progression.

The apoptotic cell death is accompanied by the depolarization of mitochondrial membrane, decrease in MMP, and elevated ROS accretion (Kurokawa et al., 2009). The dual staining was broadly utilized to differentiate the nuclear damages and apoptotic cell death in cancer cells. The sample drugs and target agents were usually investigated by dual staining for their apoptotic stimulating capacity in cancer cells. This technique clearly differentiates the both early and late apoptotic cell deaths (Gherghi et al., 2003). Our findings from the current work revealed that the formulated CA-ZnONPs (15 and 20 µg) supplemented MG-63 cells displayed the strong orange/red fluorescence that indicates the apoptotic cell death (Fig. 10). Hence, it was clear that the CA-ZnONPs treatment provoked the apoptotic cell death in MG-63 cells. The decrease in cell adhesion, improved cell migration, degradation of intracellular matrix, invasion to other organs are interrelated with the cancer progression (Hanahan and Weinberg, 2000). The treatment with the fabricated CA-ZnONPs to MG-63 cells was demonstrated the reduced cell adhesion and it reveals that the CA-ZnONPs could disaggregate the MG-63 cells through the inhibition of cell adhesion (Fig. 11).

The elevated antibiotic resistance of pathogens and re-emergence of infectious diseases worldwide demonstrates the serious threat to the humans. The nanotechnology has evolved with the abundant benefits against the severe consequences of infectious microbial resistance towards the existing antibiotics. NPs could attach effectively on the microbial surface and break their cell wall, which further results in cell death. It was already observed that the NPs with size less than 20 nm can infiltrate into bacterial cell wall and in turn obstruct the biochemical mechanisms via the demolition of the cell organs that finally results in the bacterial death (Arakha et al., 2015). ZnONPs are extensively investigated nowadays because of their exclusive antimicrobial actions. ZnO is well known to demonstrate the photocatalytic and photooxidizing effects and regarded as biosafe. ZnONPs are reported to be more potent against the infectious pathogens in the size ranging from micro to nanometers (Sur and Mukhopadhyay, 2019). The most peculiar character of gram-positive bacteria is the breadth of the cell wall because of the peptidoglycan layer. It was also mentioned that the ZnONPs could damage the bacterial cell wall and results in lysis of intercellular organelles and eventually evidenced to be deadly for the bacteria (Abebe et al., 2020).

The antimicrobial activity of CA-ZnONPs showed the potent antimicrobial activity against the tested pathogens. The clear zones of inhibition around the CA-ZnONPs loaded wells observed (Fig. 7). The *E. coli* bacteria exhibited the more sensitivity against the CA-ZnONPs than other tested pathogens. The antimicrobial efficacy of CA-ZnONPs is normally influenced by ROS that is majorly associated to the size, huge surface area, elevation in oxygen demands and Zn^2+^ ions release (Karthikeyan et al., 2020). From antibacterial activity of CA-ZnONPs, it showed similar antibacterial effect as compared to the standard antibiotic amoxicillin. The nano sized NPs certainly has a more activity as antibacterial. The XRD pattern showed the particles size of ZnO NPs was 41.25 nm. Nano size could easily enter into bacterial cell membranes because of their huge interfacial area and PL study show singly ionized Zn vacancies (Zn_v_) and interstitial oxygen vacancies (O_i_) at 491 of the sample; because of the ROS elevating effects of the CA-ZnONPs in the bacterial cells, enhancing their antibacterial efficiency. The antibacterial efficacy of the CA-ZnONPs normally stands on the occurrence of ROS (Karthikeyan et al., 2020).

## 5 Conclusion

In summary, the synthesis of CA-ZnONPs using aqueous *C. auriculata* flower extract has been demonstrated. These CA-ZnONPs were prepared at ambient conditions with *C. auriculata* flower acting as both the reducing agents. The XRD investigation proved that the CA-ZnONPs has the hexagonal wurtzite structure. FE-SEM images, the CA-ZnONPs was exhibit flake structure and the average size of the nanoparticle 41 nm. EDAX study revealed the chemical constituents was determined for the formulated CA-ZnONPs. From UV–Vis spectra, CA-ZnONPs absorption spectrum noted at 376 nm. The photoluminescence studies were demonstrated the CA-ZnONPs band emission due to zinc vacancies, oxygen vacancies, and surface defects. CA-ZnONPs showed a similar antibacterial effect to the antibiotic amoxicillin. CA-ZnONPs notably diminished the cell viability, decreased the MMP and endorsed the apoptotic cell death in OS MG-63 cells. These outcomes evidences that the CA-ZnONPs could be a potent chemotherapeutic agent for the OS treatment in the future. The additional studies still required in the future to make a clear understanding of chemotherapeutic role of formulated CA-ZnONPs against OS.

## Declaration of Competing Interest

The authors declare that they have no known competing financial interests or personal relationships that could have appeared to influence the work reported in this paper.
